# Healthcare use among cancer survivors during the COVID-19 pandemic: results from the SHARE COVID-19 Survey

**DOI:** 10.1007/s00520-024-08885-6

**Published:** 2024-10-10

**Authors:** Ana Sofia Pimentel, Ana Rute Costa

**Affiliations:** 1 PortoEPIUnit – Instituto de Saúde Pública Universidade do Porto, Rua das Taipas N.º 135, 4050-600 Porto, Portugal; 2 Laboratório para a Investigação Integrativa e Translacional em Saúde Populacional (ITR), Rua das Taipas N.º 135, 4050-600 Porto, Portugal; 3 Departamento de Ciências da Saúde Pública e Forenses, e Educação Médica, Faculdade de Medicina da Universidade do Porto, Porto, Portugal

**Keywords:** Neoplasms, Cancer survivors, COVID-19, Healthcare surveys

## Abstract

**Purpose:**

To estimate the association between a previous cancer diagnosis and healthcare use during the COVID-19 pandemic among Europeans and Israelis individuals.

**Methods:**

This cross-sectional study was based on data from the Survey of Health, Ageing and Retirement in Europe (SHARE), including the SHARE COVID-19 Survey, which was conducted in the summer of 2020, in 27 countries. Cancer survivors (CS, *n* = 6409) were country-, sex-, age-, and education-matched (1:2) to non-cancer individuals (NC). Adjusted odds ratios (OR) and 95% confidence intervals (95%CI) were computed using logistic regression.

**Results:**

Overall, CS were more likely to refer that they forwent medical appointments due to fear of COVID-19 (OR = 1.29, 95%CI 1.19–1.41) than NC, particularly those who lived with their partner and other relatives (OR = 1.79, 95%CI 1.39–2.30). Likewise, CS had their medical appointments postponed more often (OR = 1.54, 95%CI 1.44–1.64); this association was stronger among CS who lived with their partner and other relatives (OR = 1.96, 95%CI 1.63–2.36) who reported higher economic difficulties (OR = 1.73, 95%CI 1.50–2.00) and those with no multimorbidity (OR = 1.85, 95%CI 1.62–2.11). CS were also more likely to refer that they were unable to book an appointment (OR = 1.43, 95%CI 1.26–1.63), particularly those who reported that a person close to them died due to COVID-19 (OR = 2.72, 95%CI 1.47–5.01).

**Conclusion:**

CS were more likely to forgo medical treatment, report healthcare postponements, and be unable to book an appointment than NC, which highlights the importance of closely monitoring the long-term impact of the COVID-19 pandemic along the cancer care continuum.

## Introduction

The Severe Acute Respiratory Syndrome Coronavirus 2 (SARS-CoV-2) was identified in 2019, in Wuhan, China, after a group of patients developed severe pneumonia and rapidly spread to several countries [1]. Consequently, in March 2020, the World Health Organization (WHO) declared the disease caused by this virus (named COVID-19) a pandemic [2]. As of May 2024, there were more than 775 million confirmed cases and 7 million deaths reported, with 36% and 32% of them occurring in Europe, respectively [3].

To mitigate the spread and morbidity of SARS-CoV-2, a wide range of measures were adopted, with most hospital and primary healthcare services being diverted to respond to the disease [4]. In fact, the COVID-19 pandemic led to a redefinition of “essential” care [5], with postponements and cancellations of non-urgent appointments, treatments, and surgeries, as well as a transition into virtual rather than in-person appointments [6], affecting mainly people with chronic diseases [4]. Regarding oncological diseases in particular, the WHO estimated that 42% of countries reported partial or complete disruptions in cancer treatment, and the postponement of public screening programs was also widespread, being reported by more than half of the countries [7].

The sudden shift in oncological care due to the COVID-19 pandemic may have had relevant consequences in terms of cancer diagnosis, management, and prognosis [8]. Overall, studies have shown a deleterious effect of the COVID-19 pandemic among adult populations, namely, a decrease in cancer survival [9, 10] and a higher overall short-term mortality [9]. These findings may be explained by later diagnosis and at a more advanced stage [11], as well as the suboptimal treatment of cancer patients [12]. As such, a comprehensive assessment of the use of healthcare services during this period is crucial for healthcare systems to monitor and manage healthcare provided to cancer survivors (CS) across all phases of the cancer continuum, as well as to develop plans and strategies to be implemented in future pandemic crises.

Therefore, this study aimed to quantify the association between a previous cancer diagnosis and healthcare use during the COVID-19 pandemic, in a large sample of older Europeans and Israelis, according to sociodemographic, health-related, and cancer characteristics, by comparing CS with non-cancer individuals (NC), i.e., participants without a previous diagnosis of cancer.

## Methods

The present cross-sectional study is based on data from a specific dataset of the Survey of Health, Ageing and Retirement in Europe (SHARE), addressing the COVID-19 outbreak. As previously described in detail elsewhere [13], SHARE is a multidisciplinary and cross-national panel study, which involves individuals aged ≥ 50 years, as well as their partners, independent of their age, living in 27 European countries and Israel. In most countries, the selection of participants was based on national official registers that included individuals with reliable information on age. When this information was not available, a screening procedure to identify the age of respondents was applied before beginning fieldwork. The sampling designs were drawn upon probability-based selection frameworks, with most countries using a multi-stage sampling design [14].

The SHARE project started in 2004, and to date, eight waves of data collection have been conducted. All respondents who were interviewed in any previous wave are part of the longitudinal sample. Additionally, refreshment samples are drawn regularly, first to maintain the representation of younger cohorts who were not age-eligible in previous waves and second to compensate for the reduction in the size of the sample due to deaths, illness, and refusals [15].

Due to the COVID-19 pandemic and the implementation of lockdown measures worldwide, the data collection of the Eighth Wave of SHARE was interrupted, and a new survey was developed to collect data on the same topics as in the regular SHARE questionnaire but shortened and targeted to the COVID-19 living situation (SHARE COVID-19). Assessments were mainly performed between June and August 2020, except in Austria where interviews were conducted between July and September 2020. Overall, more than 57 thousand participants were included in this specific survey [16].

### Data collection

All participants of SHARE COVID-19 were assessed through computer-assisted telephone interviews, covering the most important life domains for the target population during the lockdown—health and health behavior, mental health, infections and healthcare, changes in work and economic situation, and social networks [16].

Regarding the previous diagnoses of illnesses or health conditions, participants were asked if they had any diagnoses since the last interview, including cancer. Therefore, data from previous waves (waves 1 to 8) were also used to ensure a correct classification of all participants regarding their history of chronic conditions. For the purposes of this study, participants who reported a medical diagnosis of cancer in any SHARE wave were considered CS, independently of the time since diagnosis and the current treatment phase.

The assessment of healthcare use during the COVID-19 pandemic was conducted by asking participants whether they had been treated at a hospital or other doctor’s office or medical facility since the outbreak. Whenever applicable, the level of satisfaction with these visits/treatments and the reason for dissatisfaction were also questioned. To evaluate the effects of COVID-19 in healthcare, three questions were asked: (1) “Since the outbreak of Corona, did you forgo medical treatment because you were afraid to become infected by the Corona virus?”; [2] “Did you have a medical appointment scheduled, which the doctor or medical facility decided to postpone due to Corona?”; [3] “Did you ask for an appointment for a medical treatment since the outbreak of Corona and not get one?”. If participants answered yes to any of these questions, they were asked which type of medical treatment was affected, namely: check-up at a general practitioner; check-up at a specialist, including a dentist; a planned medical treatment, including a surgery; allied health professionals, including physiotherapy, psychotherapy, and rehabilitation; and some other type of medical treatment.

Sociodemographic data were also retrieved, namely, sex, age, country of residence, household composition, and ability to make ends meet since the outbreak. Participants were asked about their employment status before the outbreak; those who answered that they were employed were also questioned if they became unemployed, were laid off, or had to close their businesses due to the pandemic. Afterward, employment status was categorized as (1) not employed; (2) employed; and (3) unemployed, laid off, or business closed due to COVID-19. A question regarding the existence of economic difficulties since the COVID-19 outbreak was also applied: “Thinking of your household’s total monthly income since the outbreak of Corona, would you say that your household is able to make ends meet with great difficulty, with some difficulty, fairly easily, or easily?”; answers were further dichotomized as (1) easily or fairly easily and (2) with some or great difficulties.

Data from previous waves were also used to complete information on the educational level of participants. This variable was further categorized using the International Standard Classification of Education (ISCED) 1997 coding of education, ranging from 0, no education or pre-primary education, to 6, second stage of tertiary education (advanced research qualification).

Regarding participants’ health status, participants were asked if they or anyone close to them tested positive for SARS-CoV-2 and if anyone close to them died due to COVID-19. The presence of multimorbidity was defined as previous diagnoses of two or more chronic diseases, including heart attack, high blood pressure, high blood cholesterol, stroke, diabetes, chronic lung disease, stomach, duodenal or peptic ulcer, as well as Parkinson’s disease, cataracts, fractures, dementia, or other conditions.

### Participants

From a total of 57,303 individuals evaluated in SHARE COVID-19, those with no data on key variables were excluded (*n* = 1095). Afterward, CS and NC were matched 1:2 by country, sex, five-year age group (< 50, 50–54 to 80–84, and ≥ 85 years), and educational level (“Low”: ISCED-97 levels 0, 1, and 2; “Middle”: levels 3 and 4; “High”: levels 5 and 6; and “Other” (e.g., no degree yet/still in school). A total of 6409 CS and 12,818 NC were considered for data analysis.

### Statistical analysis

The effect of a previous cancer diagnosis on healthcare use during the COVID-19 pandemic was quantified through adjusted odds ratios (ORs) and the respective 95% confidence intervals (CIs), using logistic regression. All models included age (continuous variable) and educational level (seven categories defined by the ISCED). Furthermore, estimates stratified by the variables not used for the matching procedure, namely, employment status, household composition, ability to make ends meet since the outbreak, previous infection with SARS-CoV-2, and multimorbidity, also included country and sex as adjustment variables, in order to control for potential residual confounding.

Statistical analyses were performed using STATA® version 15.1 (College Station, TX: StataCorp LLC, 2017).

## Results

The participants’ sociodemographic characteristics and health status are shown in Table 1. When compared with NC, CS were less frequently employed at the beginning of the outbreak (9.7% vs. 11.6%, *p* < 0.001) and had a higher prevalence of multimorbidity (75.8% vs. 69.8%, *p* < 0.001). No statistically significant differences regarding previous infection of SARS-CoV-2 or death of anyone close to them due to COVID-19 were observed between CS and NC.

**Table 1:** Sociodemographic characteristics, health status, and cancer-related characteristics among cancer survivors and individuals without a previous diagnosis of cancer

		CS^a^ (*n* = 6409)	NC^a^ (*n* = 12,818)	
		*N* (%)	*N* (%)	*P*-value
Sociodemographic characteristics				
Sex	Female	3722 (58.1)	7444 (58.1)	
	Male	2687 (41.9)	5374 (41.9)	––^**a**^
Age (years)	< 70	2178 (34.0)	4356 (34.0)	
	70–79	2622 (40.9)	5244 (40.9)	
	≥ 80	1609 (25.1)	3218 (25.1)	––^**a**^
Education^a^	Low	2107 (32.9)	4214 (32.9)	
	Middle	2666 (41.6)	5332 (41.6)	
	High	1631 (25.4)	3262 (25.4)	
	Other	5 (0.1)	10 (0.1)	––^**a**^
Region^b^	Northern Europe	1468 (22.9)	2978 (22.9)	
	Southern Europe	1676 (26.2)	3390 (26.2)	
	Western Europe	2207 (34.4)	4448 (34.4)	
	Eastern Europe	859 (13.4)	1744 (13.4)	
	Israel	199 (3.1)	398 (3.1)	––^**a**^
Household composition	Living alone	1791 (28.0)	3512 (27.4)	
	Living with a partner only	3517 (54.9)	7046 (55.0)	
	Living with a partner and any other relatives	749 (11.7)	1546 (12.1)	
	Living with any other relatives	352 (5.5)	714 (5.6)	0.797
Employment status	Not employed	5635 (88.1)	10,970 (85.7)	
	Employed	620 (9.7)	1487 (11.6)	
	Unemployed, laid off or business closed due to COVID-19	143 (2.2)	350 (2.7)	** < 0.001**
Household’s ability to make ends meet since the outbreak	Easily or fairly easily	3187 (70.7)	6307 (71.7)	
	With some or great difficulties	1322 (29.3)	2492 (28.3)	0.228
Health status				
SARS-CoV-2 infection	No	5832 (91.7)	11,730 (92.4)	
	Yes, anyone close	495 (7.8)	905 (7.1)	
	Yes, the participant	32 (0.5)	66 (0.5)	0.257
Death of anyone close due to SARS-CoV-2 infection	Yes	205 (3.2)	358 (2.8)	0.114
Multimorbidity	Yes^c^	4857 (75.8)	8949 (69.8)	** < 0.001**

*CS*, cancer survivors; *NC*, non-cancer individuals. ^a^CS were matched 1:2 to NC, by country, sex, five-year age group (< 50, 50–54 to 80–84, and ≥ 85 years), and educational level (“Low”: ISCED-97 levels 0, 1, and 2; “Middle”: levels 3 and 4; “High”: levels 5 and 6; and “Other” (e.g., no degree yet/still in school). ^b^ “Northern Europe” includes Denmark, Estonia, Finland, Latvia, Lithuania, and Sweden; “Southern Europe” includes Croatia, Cyprus, Greece, Italy, Portugal, Slovenia, and Spain; “Western Europe” includes Austria, Belgium, France, Germany, Luxembourg, Netherlands, and Switzerland; “Eastern Europe” includes Bulgaria, Czech Republic, Hungary, Poland, and Romania. ^c^Multimorbidity was defined as previous diagnoses of ≥ 2 chronic diseases, including heart attack; high blood pressure; high blood cholesterol; stroke and diabetes; chronic lung disease; stomach, duodenal, or peptic ulcer; Parkinson’s disease; cataracts; fractures; dementia or other conditions. Note: The total may not add to the sample size of each group due to missing values. Bold values represent statistically significant differences between groups (*p* < 0.05)

CS reported a higher use of hospital care since the beginning of the SARS-CoV-2 outbreak, when compared with NC (16.9% vs. 8.3%, *p* < 0.001), as well as a greater use of other medical facilities (38.2% vs. 30.7%, *p* < 0.001). No statistically significant differences were observed between CS and NC regarding the use of hospital or other medical care since the outbreak due to the SARS-CoV-2 infection or satisfaction with the way they were treated (Table 2).

**Table 2:** Healthcare use during the COVID-19 pandemic and satisfaction with the healthcare received among cancer survivors and individuals without a previous diagnosis of cancer

			CS^a^ (*n* = 6409)	NC^a^ (*n* = 12,818)	
			*N* (%)	*N* (%)	*P*-value
A. Hospital care	**Use during the SARS-CoV-2 outbreak**	Yes	1082 (16.9)	1062 (8.3)	** < 0.001**
	**Use related with COVID-19**	Yes	9 (0.1)	20 (0.2)	0.792
	**Satisfaction** with the way they were treated^a^	Somewhat or very satisfied	1017 (94.9)	998 (94.7)	
		Somewhat or very dissatisfied	55 (5.1)	56 (5.3)	0.850
	**Reasons for being dissatisfied** with the way they were treated^b^	Long wait time	17 (30.9)	19 (33.9)	0.734
		Overcrowded	6 (10.9)	6 (10.7)	0.974
		Doctor or nurses did not have time	26 (47.3)	28 (50.0)	0.774
		Shortage of equipment and supplies	2 (3.6)	1 (1.8)	0.548
		Insufficient safety measures against infections	4 (7.3)	4 (7.1)	0.979
		Other	30 (54.6)	31 (55.4)	0.932
B. Other medical facility	**Use during the SARS-CoV-2 outbreak**	Yes	2446 (38.2)	3929 (30.7)	** < 0.001**
	**Use related with COVID-19**^a^	Yes	46 (1.9)	77 (2.0)	0.824
	**Satisfaction** with the way they were treated^a^	Somewhat or very satisfied	1792 (72.9)	2891 (73.3)	
		Somewhat or very dissatisfied	77 (3.2)	110 (2.8)	0.428
	**Reasons for being dissatisfied** with the way they were treated^b^	Long wait time	18 (23.4)	26 (23.6)	0.967
		Overcrowded	4 (5.2)	6 (5.4)	0.938
		Doctor or nurses did not have time	31 (40.3)	29 (26.4)	**0.045**
		Shortage of equipment and supplies	4 (5.2)	3 (2.7)	0.382
		Insufficient safety measures against infections	7 (9.1)	7 (6.4)	0.486
		Other	45 (58.4)	74 (67.3)	0.217

*CS*, cancer survivors; *NC*, non-cancer individuals. ^a^Only among those who used hospital care during the SARS-CoV-2 outbreak. ^b^Only among those who were somewhat or very dissatisfied with the way they were treated during the SARS-CoV-2 outbreak. Note: Bold values represent statistically significant differences between groups (*p* < 0.05)

**Fig. 1:**
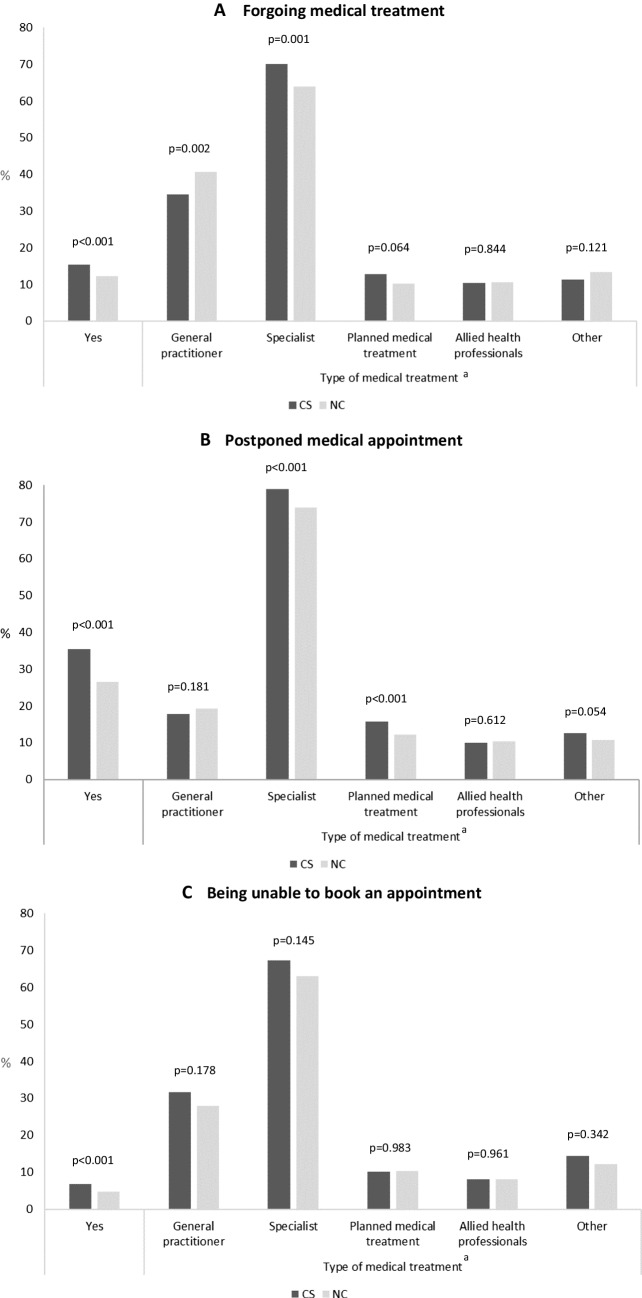
Differences in healthcare use during the COVID-19 pandemic among cancer survivors and individuals without a previous diagnosis of cancer. **A** Forgoing medical treatment. **B** Postponed medical appointment. **C** Being unable to book an appointment. *CS*, cancer survivors; *NC*, non-cancer individuals. ^a^Only among participants who answered “yes” to the respective problem with healthcare use. Type of medical treatment includes check-up at a general practitioner; check-up at a specialist, including a dentist; a planned medical treatment, including an operation; physiotherapy, psychotherapy, and rehabilitation; and some other type of medical treatment

Overall, CS more frequently referred that they forwent medical treatment since the start of the COVID-19 outbreak due to fear of becoming infected with SARS-CoV-2 (15.4% vs. 12.4%, *p* < 0.001), particularly appointments with a specialist or a dentist check-up (70.3% vs. 63.7%, *p* = 0.001); on the contrary, they forwent general practitioner check-ups less frequently (34.4% vs. 40.5%, *p* = 0.002). When compared with NC, the medical appointments for CS were postponed more often by health providers due to the outbreak (35.6% vs. 26.5%, *p* < 0.001), namely, those with a specialist or a dentist (79.0% vs. 74.0%, *p* < 0.001) and planned medical treatment or surgery (15.8% vs. 12.3%, *p* < 0.001). Additionally, being unable to book an appointment for medical treatment since the start of the outbreak was more frequent among CS than NC (6.8% vs. 4.8%, *p* < 0.001) (Fig. 1).

**Fig. 2:**
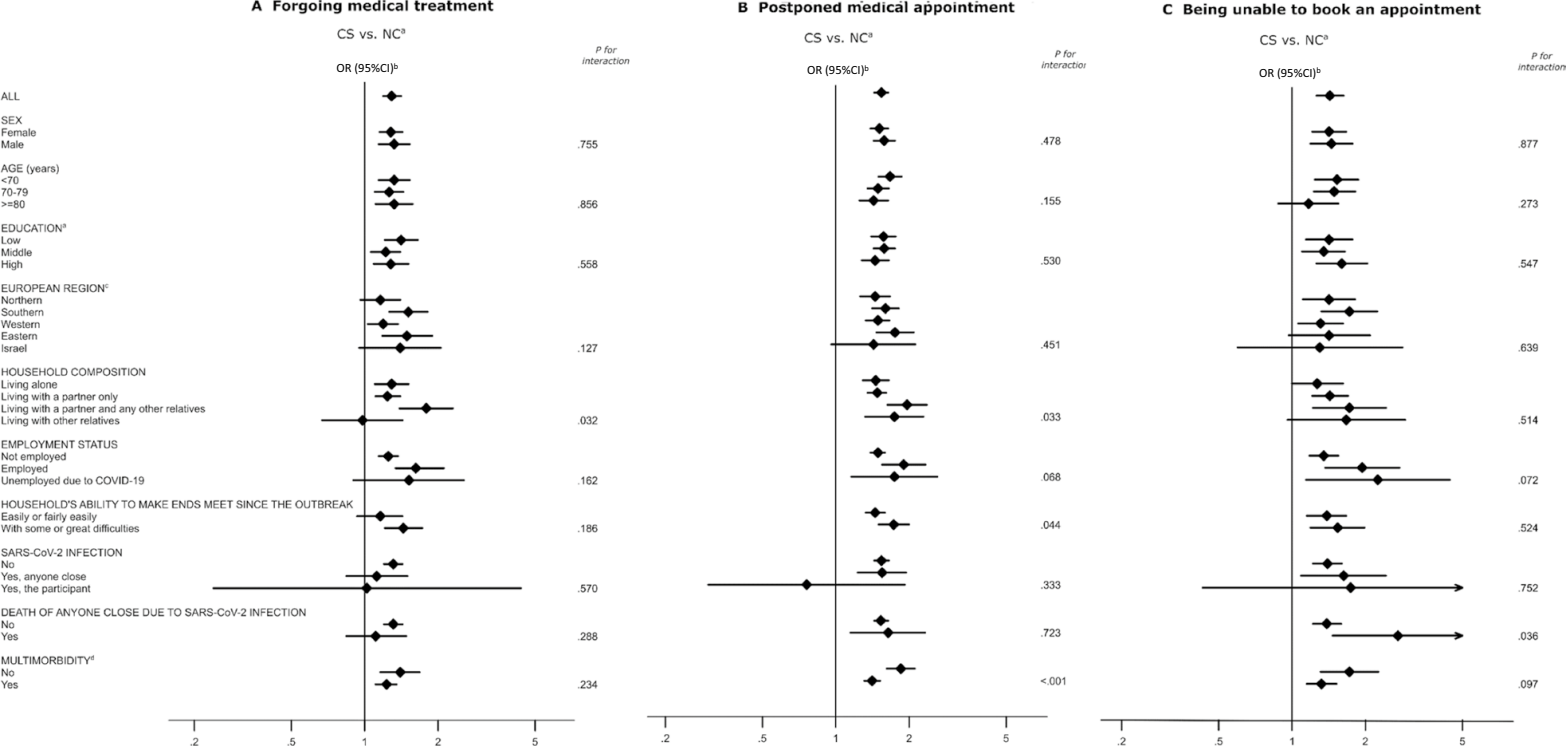
Healthcare use during the COVID-19 pandemic among cancer survivors and individuals without a previous diagnosis of cancer, according to sociodemographic characteristics and health status. *CI*, confidence interval; *CS*, cancer survivors; *NC*, non-cancer individuals; *OR*, odds ratio. ^a^CS were matched 1:2 to NC, by country, sex, five-year age group (< 50, 50–54 to 80–84, and ≥ 85 years), and educational level (“Low”: ISCED-97 levels 0, 1, and 2; “Middle”: levels 3 and 4; “High”: levels 5 and 6; and “Other” (e.g., no degree yet/still in school). ^b^All models were adjusted for age (continuous variable) and educational level (seven categories defined by the ISCED). Employment status, household composition, ability to make ends meet since the outbreak, previous infection with SARS-CoV-2, and multimorbidity also included country and sex as adjustment variables. ^c^ “Northern Europe” includes Denmark, Estonia, Finland, Latvia, Lithuania, and Sweden; “Southern Europe” includes Croatia, Cyprus, Greece, Italy, Portugal, Slovenia, and Spain; “Western Europe” includes Austria, Belgium, France, Germany, Luxembourg, Netherlands, and Switzerland; “Eastern Europe” includes Bulgaria, Czech Republic, Hungary, Poland, and Romania. ^d^Multimorbidity was defined as previous diagnoses of ≥ 2 chronic diseases, including heart attack; high blood pressure; high blood cholesterol; stroke and diabetes; chronic lung disease; stomach, duodenal, or peptic ulcer; Parkinson’s disease; cataracts; fractures; dementia; or other conditions

CS were more likely to refer that they forwent medical appointments due to fear of becoming infected with SARS-CoV-2 (OR = 1.29, 95%CI 1.19–1.41). This association was stronger among those who lived with their partner and any other relatives (OR = 1.79, 95%CI 1.39–2.30; *p* for interaction = 0.032). Additionally, a tendency towards higher odds for forgoing medical appointments was observed among CS from Southern and Eastern Europe (OR = 1.51, 95%CI 1.26–1.81; OR = 1.49, 95%CI 1.18–1.89, respectively; *p* for interaction = 0.127), among employed individuals (OR = 1.62, 95%CI 1.24–2.11; *p* for interaction = 0.162) and those with greater economic difficulties (OR = 1.44, 95%CI 1.21–1.72; *p* for interaction = 0.186) (Fig. 2).

Appointment postponements by health providers due to the pandemic were reported more often by CS than NC (OR = 1.54, 95%CI 1.44–1.64), which was particularly observed among those who lived with their partner and other relatives (OR = 1.96, 95%CI 1.63–2.36; *p* for interaction = 0.033), who reported greater economic difficulties due to the pandemic (OR = 1.73, 95%CI 1.50–2.00; *p* for interaction = 0.044) and those with no multimorbidity (OR = 1.85, 95%CI 1.62–2.11; *p* for interaction < 0.001). Furthermore, this outcome tended to be more frequently reported among younger (OR = 1.67, 95%CI 1.50–1.88; *p* for interaction = 0.155) and employed individuals (OR = 1.90, 95%CI 1.55–2.33; *p* for interaction = 0.068) (Fig. 2).

Regarding being unable to book an appointment for medical treatment, CS reported this problem more frequently than NC (OR = 1.43, 95%CI 1.26–1.63); this association was stronger among those who reported that a person close to them died due to COVID-19 (OR = 2.72, 95%CI 1.47–5.01; *p* for interaction = 0.036). A tendency for higher odds was observed among younger individuals (OR = 1.53, 95%CI 1.24–1.87; *p* for interaction = 0.273), employed and unemployed due to the outbreak (OR = 1.94, 95%CI 1.37–2.76; OR = 2.25, 95%CI 1.14–4.44, respectively; *p* for interaction = 0.072), and those with no multimorbidity (OR = 1.72, 95%CI 1.31–2.26; *p* for interaction = 0.097) (Fig. 2).

## Discussion

This study has shown that CS have an increased use of healthcare services during the COVID-19 pandemic in comparison with NC, namely, more frequent contact with hospital care and other medical facilities. No statistically significant differences were observed regarding the satisfaction of the treatment received. Additionally, CS forwent medical treatment due to fear of becoming infected with SARS-CoV-2 more often, and they were also more likely to report postponements of medical appointments and to be unable to book an appointment for medical treatment. These outcomes were particularly observed among individuals who lived with their partner and any other relatives, who reported greater economic difficulties due to the pandemic, with no multimorbidity, as well as among those who reported that a person close to them died due to COVID-19.

Previous studies [17–19] have shown that CS use healthcare services more frequently than the general population, which was also observed in the present work. CS often experience poorer health and wellbeing, due to the cancer and its treatments [20], and have a higher risk of developing a new cancer [21], which may contribute to their greater use of healthcare services, even during the pandemic. Additionally, CS under active cancer treatment report a higher use of healthcare services [22]. However, information regarding current cancer treatment was not available in the current study. Furthermore, in the present study, we did not find differences between CS and NC regarding satisfaction with how participants were treated. In fact, this question was only asked among those who used healthcare services. As such, future studies should assess the overall satisfaction with healthcare provided during the pandemic, since those who forwent, postponed, or were unable to book an appointment may be more dissatisfied.

The COVID-19 pandemic affected health systems worldwide and resulted in the disruption of routine care in many clinical services with particular repercussions on cancer care, including the postponement of consultations and treatments [23]. In fact, despite the higher use of healthcare reported by CS than NC, they also forwent medical appointments due to fear of becoming infected by SARS-CoV-2. This result is in line with a prior study that found that CS were more likely to cancel appointments or treatments scheduled at the hospital [24]. Cancer history, including immunocompromised state, advanced age, and poor functional status, may contribute to a higher risk for severe complications and poorer outcomes due to COVID-19 observed among these patients [25], including a more frequent need for mechanical ventilation or admission to an intensive care unit or even death due to COVID-19. Likewise, it has been reported that patients with cancer appear to have an increased risk of contracting SARS-CoV-2, compared with patients without cancer [26]. Furthermore, since CS use healthcare more frequently, it may also be expected that the postponements and cancellations observed during the pandemic will be more reflected in this group of patients. This finding is particularly relevant since interruptions of treatments or sub-optimally delivered oncology therapy, as well as the postponement of surgeries and reduced follow-up [27], may have severe consequences, including increased mortality [10, 28].

We found that the association of a previous cancer diagnosis with the occurrence of appointment postponements was stronger among younger CS and those with no multimorbidity. Younger people tend to be less resistant to applying the recommended sanitary measures, which results in higher compliance with personal protective behaviors [29]. Additionally, COVID-19 has more severe implications for CS than the general population [30]. As such, it is expected that CS at a younger age may comply more strictly with the recommended standards and have a higher perception of their risks, which may lead to an increased fear of being infected in hospital settings, and forgoing scheduled appointments and treatments. Furthermore, younger CS and those with no multimorbidity may perceive themselves as less severe cases and, therefore, may feel less need to keep appointments on scheduled dates.

A previous study conducted by our research team found a stronger association between a previous cancer diagnosis and healthcare use among CS living with other relatives besides their partners [17]. As such, it was not surprising that these patients were the most affected by the COVID-19–related changes in healthcare use. Additionally, we cannot exclude the hypothesis that patients with a higher number of household members may have a greater fear of infecting their relatives. Likewise, the CS with greater economic difficulties canceled and postponed their appointments more often, which may be related to the fear of calling out of work and, consequently, losing their job during the pandemic [31].

Countries from Southern Europe were particularly affected by the pandemic, with a higher incidence of COVID-19 observed in these countries, as well as the application of more restrictive measures to mitigate the SARS-CoV-2 outbreak, including longer periods of lockdowns. These facts may partially justify the tendency for CS living in Southern and Eastern Europe forwent medical appointments more frequently.

### Strengths and limitations

The present study is based on a large sample of European and Israeli citizens, which assessed a wide range of characteristics, including sociodemographic and health status. Furthermore, the SHARE COVID-19 Survey allows for a detailed description of the impact of the first wave of the COVID-19 pandemic, which has severely affected all health systems around the world. Furthermore, we used different approaches for minimizing selection bias and confounding, namely, matching and adjustment for a large number of potential confounders, and we also analyzed the potential interaction of different variables on the association between a previous cancer diagnosis and healthcare use during the COVID-19 pandemic.

Nevertheless, there are some limitations that should be pointed out. A previous diagnosis of cancer and healthcare use were self-reported, which may have contributed to an underestimation of the magnitude of the observed effects. In fact, patients tend to report less medical care use when compared with data retrieved from computerized clinical records [32], and this effect may be particularly stronger among CS since they usually have a higher use of healthcare.

SHARE data collection is usually collected through computer-assisted personal interviewing, while this latest questionnaire was administered via telephone. On the one hand, telephone interviews may reduce response bias that can occur with in-person interviews because of the anonymity of the telephone and the absence of an interviewer in person; on the other hand, less information is reported, and there is a challenge to maintain the participant involved in the questionnaire [33]. Nevertheless, it is not expected that the information used in the present study is affected by the method of data collection in any significant manner.

Another limitation is related to the cross-sectional design of the present study, which may have led to an underrepresentation of CS with worse disease conditions, including with a lower survival; consequently, this may have contributed to underestimating healthcare use among CS.

Furthermore, since this questionnaire was applied in the first months after the outbreak, we may hypothesize that the negative impact of COVID-19 may be stronger with the evolution of the pandemic and the application of new restrictions in the following COVID-19 waves.

## Conclusion

A previous cancer diagnosis was associated with an increased use of healthcare during the COVID-19 pandemic, as well as with a more frequent report of appointment cancelations, postponements, or denials, among older Europeans and Israeli individuals. The first months of the COVID-19 pandemic led to changes in healthcare provided to CS, including treatment and follow-up, which may have a deleterious impact on the care and prognosis of CS. Additionally, it is expected that these difficulties in oncological care worsen in the next few years due to the backlog of CS needing assessment along with the expected normal and routine volume of CS and the ongoing reallocation of resources. As such, the results of the present study highlight the importance of developing a strategic prioritization of care and closely monitoring the long-term impact of the COVID-19 pandemic on the cancer care continuum.

## Data Availability

The SHARE data that support the findings of this study are available from the SHARE Research Data Center. Restrictions apply to the availability of these data, which were used under license for this study. Data are available at http://www.share-project.org with the permission of the SHARE Research Data Center.
